# Gene therapy and its implications in Periodontics

**DOI:** 10.4103/0972-124X.51886

**Published:** 2009

**Authors:** Swapna Mahale, Nitin Dani, Shumaila S. Ansari, Triveni Kale

**Affiliations:** *Professor and Guide, MGV's KBH Dental College and Hospital, Panchavati, Nashik - 422 003, Maharashtra, India*; 1 *Professor and HOD, MGV's KBH Dental College and Hospital, Panchavati, Nashik - 422 003, Maharashtra, India*; 2 *PG Student, MGV's KBH Dental College and Hospital, Panchavati, Nashik - 422 003, Maharashtra, India*; 3 *Lecturer, MGV's KBH Dental College and Hospital, Panchavati, Nashik - 422 003, Maharashtra, India*

**Keywords:** Cell/gene therapy, growth factor biology, material science

## Abstract

Gene therapy is a field of Biomedicine. With the advent of gene therapy in dentistry, significant progress has been made in the control of periodontal diseases and reconstruction of dento-alveolar apparatus.

Implementation in periodontics include:

-As a mode of tissue engineering with three approaches: cell, protein-based and gene delivery approach.

-Genetic approach to Biofilm Antibiotic Resistance.

Future strategies of gene therapy in preventing periodontal diseases:

-Enhances host defense mechanism against infection by transfecting host cells with an antimicrobial peptide protein-encoding gene.

-Periodontal vaccination.

Gene therapy is one of the recent entrants and its applications in the field of periodontics are reviewed in general here.

## INTRODUCTION

Genes are specific sequences of bases present on the chromosomes that form the basic unit of heredity. Each person's genetic constitution is different and the changes in the genes determine the differences between individuals. Some changes, usually in a single gene, may cause serious diseases. More often, gene variants interact with the environment to predispose some individuals to various ailments.[1]

**Figure 1:**
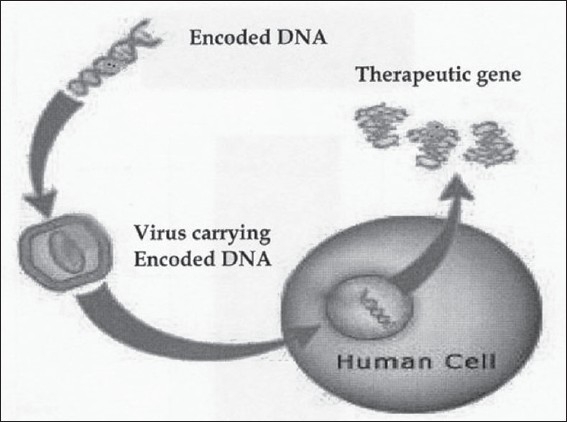
Gene therapy

Gene therapy uses purified preparations of a gene or a fraction of a gene, to treat diseases [Figure 1]. A common approach in gene therapy is to identify a malfunctioning gene and supply the patient with functioning copies of that gene. Whichever approach is used, the aim of gene therapy is to introduce therapeutic material into the target cells, where it becomes active and exerts the intended therapeutic effect.

In the mid 1980s, the focus of gene therapy was entirely on treating diseases caused by single-gene defects. However, in the late 1980s and early 1990s, the concept of gene therapy was being increasingly considered for the treatment of a number of acquired diseases.

## REVIEW OF LITERATURE

1. In 1995, the potential impact of gene therapy on dentistry was described.
2. In 2000, the first report of a fully successful gene therapy treatment—a French study involving a severe combined immunodeficiency in young children was published.
3. Wikesjö UM, Sorensen RG, Kinoshita A, Jian Li X, Wozney JM in 2004 showed the effect of rhBMP-12 on regeneration of alveolar bone and periodontal attachment.[2]
4. Gonçalves PF, Lima LL, Sallum EA, Casati MZ, Nociti FH Jr demonstrated that root cementum may modulate the expression of growth and mineral-associated factors during periodontal regeneration.[3]
5. Lin Z, Sugai JV, Jin Q, Chandler LA, Giannobile WV in 2008 demonstrated that gene delivery of PDGF-B displays sustained signal transduction effects in human gingival fibroblasts that are higher than those conveyed by treatment with recombinant human platelet-derived growth factor-BB protein.[4]

## DISCUSSION

Remarkable progress has been made in the field of gene therapy, including seven areas relevant to dental practice: bone repair, salivary glands, autoimmune disease, pain, DNA vaccinations, keratinocytes and cancer.[5]

For correcting faulty genes, one of the several approaches may be employed:[6]

- A normal gene may be inserted into a nonspecific location within the genome to replace a nonfunctional gene; this is the most common approach.
- An abnormal gene may be swapped for a normal gene through homologous recombination.
- The abnormal gene could be repaired through selective reverse mutation, which returns the gene to its normal functional status.
- The regulation (the degree to which a gene is turned on or off) of a particular gene could be altered.

### Viral approaches

**Figure 2:**
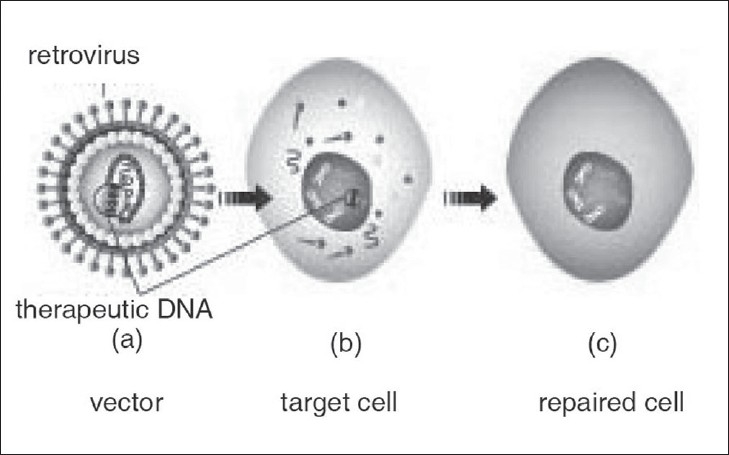
Viral vector

A gene inserted directly into a cell usually does not function. Instead, a carrier called a vector is used to introduce the therapeutic gene into the patient's target cells[7] [Figure 2]. The most common vector used is a virus.

Viral vectors are natural infectious agents for transferring genetic information. They are quite efficient, and at present they generally provide more pre-clinical and clinical utility than non-viral vectors, although that gap is diminishing.

Viruses cause diseases in humans by encapsulating and delivering the genes into cells. Some types of viruses, such as retroviruses, integrate their genetic material (which can be manipulated to include the therapeutic gene) into a chromosome in the human cell. Other viruses, such as adenoviruses, introduce their DNA into the nucleus of the cell, but the DNA is not integrated into a chromosome.[8–10,15]

Some of the different types of viruses used as vectors in gene therapy are:[11]

- Retroviruses (e.g. HIV): A class of viruses that can create double-stranded DNA copies of their RNA genomes. These copies of its genome can be integrated into the chromosomes of host cells.
- Adenoviruses: A class of viruses with double-stranded DNA genomes; they cause respiratory, intestinal and eye infections in humans.
- Adeno-associated viruses: A class of small, single-stranded DNA viruses that can insert their genetic material at a specific site on chromosome 19.
- Herpes simplex viruses: A class of double-stranded viruses that can infect a particular cell type, i.e., neurons.
- Lentiviruses and hybrid viruses (which combine the positive features of more than one virus).

The vector can be given intravenously or injected directly into a specific tissue in the body. Alternatively, cultured cells are exposed to the vector and then reintroduced into the patient.

### Non-viral approaches

Besides virus-mediated gene-delivery systems, there are several non-viral options for gene delivery.

a) The simplest method is the direct introduction of therapeutic DNA into target cells.

Disadvantage: It requires large amounts of DNA to bring out the desired effect and hence this technique has restricted use.

b) Another non-viral approach involves the creation of an artificial lipid sphere (a liposome) with an aqueous core. This liposome, which carries the therapeutic DNA, is capable of transporting the DNA through the target cell's membrane. Therapeutic DNA can also be introduced into target cells by chemically linking the DNA to a molecule that will bind to special cell receptors. Once bound to these receptors, the therapeutic DNA constructs are engulfed by the cell membrane and passed into the interior of the target cell.

Disadvantage: This delivery system tends to be less effective than the others

c) Experiments with the introduction of a 47^th^ chromosome (an artificial, human techno-chromosome) into target cells are being carried out.[12] This chromosome would exist autonomously alongside the standard 46^th^ chromosome, without affecting their functions or causing any mutations. It would be a large vector capable of carrying substantial amounts of genetic code and, because of its construction and autonomy, the body's immune system would not attack it.

Disadvantage: Difficulty in delivering such a large molecule into the nucleus of a target cell.

### Somatic and germ line gene therapy

Gene therapy can target somatic (body) or germ (egg and sperm) cells. In somatic gene therapy the recipient's genome is changed, but the change is not passed on to the next generation; with germ line gene therapy, the newly introduced gene is passed on to the offspring.[13]

### Technical difficulties in gene therapy

#### a) Gene delivery

Successful gene delivery is not easy or predictable, even in single-gene disorders. For example, although the genetic basis of cystic fibrosis is well known, the presence of mucus in the lungs makes it physically difficult to deliver genes to the target lung cells. Delivery of genes for cancer therapy may also be complicated by the disease at several sites.

#### b) Durability and integration

Some gene therapy approaches aim at long-term effects. Two possible ways of achieving this are to either use multiple rounds of gene therapy or integrate the therapeutic genes so that they remain active for some time. Integrating therapeutic DNA may cause possible undesirable side effects.

For instance, babies with the X-SCID syndrome go on to develop leukemia-like symptoms[14] as therapeutic material might have integrated at a site where it affected another gene which may have produced the rapid growth of cancerous cells.

Other approaches seek more immediate effects where integration is not the aim. For instance, in gene therapy to treat cancer, the aim may be to use ‘suicide’ genes to kill cancerous cells as quickly as possible.

#### c) Immune response

Viral vector may be recognized as ‘foreign’ and mobilize the immune system to attack it. This may hamper the efficacy of gene therapy or induce serious side effects.

#### d) Safety of vectors

Viruses present a variety of potential problems to the patient, e.g., toxicity, immune and inflammatory responses and gene control and targeting issues. In addition, viral vector may recover its ability to cause disease.

First, and thus far only, death as a result of a clinical gene transfer procedure, occurred in 1999.[1]

### Gene therapy in periodontics

The tissue engineering approach reconstructs the natural target tissue by combining four elements, namely, the scaffold, signaling molecules, blood supply and cells. Currently, genetic principles are being applied along with tissue engineering for periodontal rehabilitation.

The three basic approaches in tissue engineering are[16] the following

**Figure 3:**
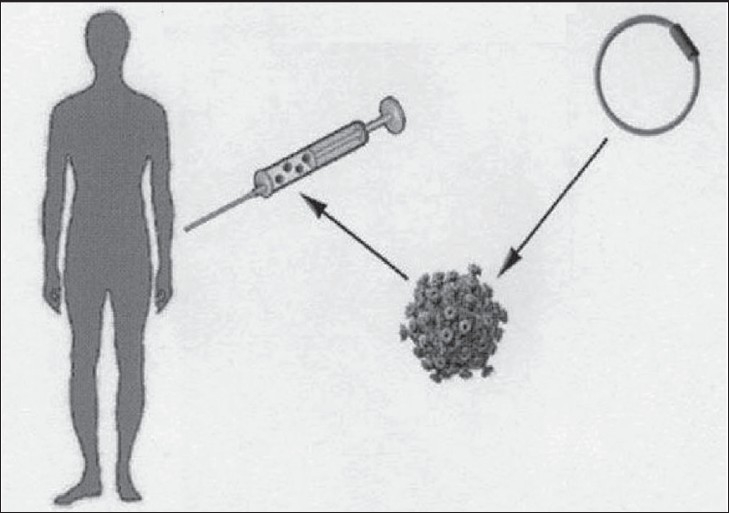
*In-vivo* gene delivery

1. ***Protein-based approach:*** Trials have been conducted using TGF-β, BMP-2, 6, 7, 12, bFGF, VEGF and PDGF.[17]
2. ***Cell-based approach:*** Several preclinical studies using mesenchymal stem cells are done. Cell-mediated gene transfer can be utilized for growth factor delivery to signaling receptors of transplanted cells (autocrine effect) and host mesenchymal cells (paracrine effect). Skeletal muscle derived cells can be used for delivery of BMP-2 and a possible source of inducible osteoprogenitor cells to participate in bone formation.[18]
3. ***Gene-delivery approach:***[19] This approach involves two basic modalities:In vivo gene delivery: In this approach, gene constructs such as plasmid DNA or a viral particle, are physically entrapped within a scaffold or a matrix [Figure 3]. When the scaffold containing the gene constructs is implanted into the tissue defect, the host cells migrate into the implant, take up the gene constructs and start producing the encoded protein.

**Figure 4:**
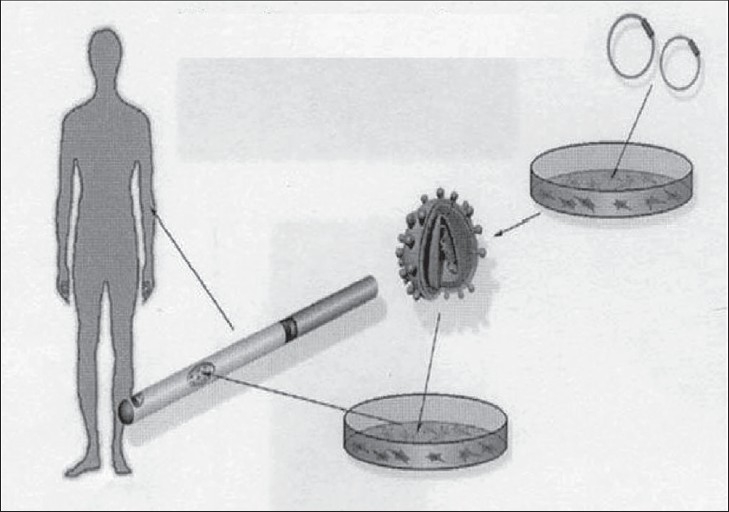
*Ex-vivo* gene delivery

*Ex vivo* gene delivery: In this approach, cultured cells are transfected (in non-viral delivery systems) or transduced (in viral delivery systems) with gene constructs in vitro before they are transplanted into the tissue defect [Figure 4].

### Clinical trials using gene therapy

#### Platelet-derived growth factor (PDGF) gene delivery

- Direct *in-vivo* gene transfer of PDGF-B stimulated tissue regeneration in large periodontal defects.[20]
- In an ex-vivo investigation, it was shown that the expression of PDGF genes was prolonged for up to 10 days in gingival wounds.[21]
- Different mechanisms of drug delivery and novel approaches to reconstruct and engineer oral- and tooth-supporting structures, namely the periodontium and alveolar bone were reviewed.[22]

#### Bone morphogenetic protein delivery

- *In vitro* and *in vivo* Ad gene transfer of BMP-7 for bone formation has been investigated.[23]
- Direct *in vivo* gene delivery of Ad/BMP-7 in a collagen gel carrier promoted successful regeneration of alveolar bone defects around dental implants.[24]

#### Some of the ongoing clinical trials in the field of medicine:[25,26]

- Gene therapy and chemotherapy in treating patients with advanced solid tumors or non-Hodgkin's lymphoma. Conditions: Adult Brain Tumor; adult non-Hodgkin's lymphoma; adult solid tumor.
- Stem cell gene therapy to treat X-SCID.
  Condition: Severe combined immunodeficiency.
- Gene therapy to prevent cancer in patients with premalignant carcinoma of the oral cavity or pharynx. Conditions: Lip and oral cavity cancer; oropharyngeal cancer.

### Recent gene-therapeutic advances in the field of implants

*BMP gene delivery for alveolar bone engineering at dental implant defects*:[27]

- Tooth loss, often a consequence of trauma or disease, can lead to the destruction of nearly half of the original tooth-supporting (or alveolar) bone.[28]
- A challenge in the tissue engineering of alveolar bone surrounding oral or dental implants is achieving the targeted and sustained delivery of growth-promoting molecules at the osteotomy site. BMP-7 (also known as osteogenic protein-1) has demonstrated the ability to stimulate bone regeneration in multiple skeletal sites, including the craniofacial complex.
- Some BMPs also participate in the development and repair of extra-skeletal tissues and organs such as the brain, kidney, and nerves.[29]
- Treatment of titanium dental implant fixtures with Ad/BMP-7 resulted in enhancement of alveolar bone defect fill, coronal new bone formation, and new bone-to-implant contact in 44 Sprague-Dawley rats.
- BMP-7-treated implants revealed a more mature lamellar bone formation, while Ad/Luc-treated defects contained mostly immature woven bone. Osseointegration as shown by SEM could be clearly seen.

An adenoviral vector with a collagen matrix to immobilize the transgene at the dental implant defect site was used that led to optimal effects in both transduction efficiency and bone repair.[17] In addition, previous studies used a similar dosing.[20] However, future dose levels using direct gene delivery of BMPs need to be explored. This technique efficiently delivered platelet-derived growth factor genes to tooth-supporting structures.[31] Peak gene expression was found one to seven days after delivery, with a subsequent decrease in expression over time. A low level of transgene expression was detected for up to 10-35 days, suggesting that the collagen was capable of immobilizing the virus for extended periods of time.[30]

### Future strategies of gene therapy in preventing periodontal diseases

#### 1. Genetic approach to biofilm antibiotic resistance:[31]

Biofilms are surface-attached microbial communities with characteristic architecture and phenotypic and biochemical properties distinct from their free-swimming, planktonic counterparts. One of the best-known of these biofilm-specific properties is the development of antibiotic resistance that can be up to 1,000-fold greater than planktonic cells. A genetic determinant of this high-level resistance in the Gram-negative opportunistic pathogen, *Pseudomonas aeruginosa* was reported. A mutant of *P. aeruginosa* capable of forming biofilms with the characteristic *P. aeruginosa* architecture, does not develop high-level biofilm-specific resistance to three different classes of antibiotics. The locus identified ndvB, is required for the synthesis of periplasmic glucans. These periplasmic glucans interact physically with tobramycin suggests that these glucose polymers may prevent antibiotics from reaching their sites of action by sequestering these antimicrobial agents in the periplasm. Biofilms themselves are not simply a diffusion barrier to these antibiotics, but rather that bacteria within these microbial communities employ distinct mechanisms to resist the action of antimicrobial agents.

#### 2. Gene therapeutics-periodontal vaccination

Host responses to recombinant hemagglutinin B of *Porphyromonas gingivalis* in an experimental rat model:[32]

Extensive studies[33,34] described the cloning of four *P. gingivalis* genes and their expression in *Escherichia coli*. These genes encode for the proteins Hag A, B, C, and D. Hag A and D have about 73.8% identity, whereas Hag B and C are 98.6% homologous. However, neither shows significant homology to Hag A. There are several genes encoding for hemagglutinin molecules, which may be an indication of their importance in virulence. Currently, Hag A and B have been more extensively characterized than Hag C or D. Furthermore, there has been a great deal of interest in the potential utilization of Hag B in vaccine development. For instance, *hagB* gene was expressed in an avirulent strain of *S. typhimurium*.[35]

## CONCLUSION

Repair of tooth supporting alveolar bone defects caused by periodontal and peri-implant tissue destruction is a major goal of reconstructive therapy. Oral and craniofacial tissue engineering has been achieved with limited success by the utilization of a variety of approaches such as cell-occlusive barrier membranes, bone substitutes and autogenous block grafting techniques. Signaling molecules such as growth factors have been used to restore lost tooth support because of damage by periodontal disease or trauma. This article reviewed emerging periodontal therapies in the areas of material science, growth factor biology and cell/gene therapy. Results from preclinical and clinical trials have been reviewed using the topical application of bone morphogenetic proteins and platelet-derived growth factor for periodontal and peri-implant regeneration. It concludes with recent research on the use of *ex vivo* and *in vivo* gene delivery strategies via gene therapy vectors encoding growth promoting and inhibiting molecules (PDGF, BMP, noggin and others) to regenerate periodontal structures including bone, periodontal ligament and cementum.
